# Cross-cultural adaptation and psychometric properties of the Myanmar version of the scale of oral health outcomes for 5-year-old children

**DOI:** 10.1371/journal.pone.0282880

**Published:** 2023-03-22

**Authors:** Saw Nay Min, Duangporn Duangthip, Sherry Shiqian Gao, Palinee Detsomboonrat

**Affiliations:** 1 Department of Community Dentistry, Faculty of Dentistry, Chulalongkorn University, Bangkok, Thailand; 2 Restorative Dental Sciences, Faculty of Dentistry, The University of Hong Kong (HKU), Hong Kong, China; 3 Department of Stomatology, School of Medicine, Xiamen University, Xiamen, China; State University of Ponta Grossa: Universidade Estadual de Ponta Grossa, BRAZIL

## Abstract

**Objective:**

The aim of this study was to cross-culturally adapt the child’s self-report and parental report of the scale of oral health outcomes for 5-year-old children (SOHO-5) for use in Myanmar (Burmese-speaking) population and to assess the reliability and validity of the Myanmar version.

**Materials and methods:**

The forward-backward translation method was used to develop the Myanmar SOHO-5 version and the final questionnaires were tested on 173 five years old children and their parents for reliability and validity. A single dentist examined the caries experience of the children (Kappa:0.90). The structural validity was assessed through confirmatory factor analysis. The internal consistency and test-retest reliability (1–2 weeks) were evaluated using Cronbach’s alpha and intraclass correlation coefficient (ICC), respectively. The association between SOHO-5 scores and additional global rating questions for child oral health status (convergent validity) and the differences between the total SOHO-5 score of children with caries and children without caries (discriminant validity) were investigated.

**Results:**

A confirmatory factor analysis indicated a good fit for the one-factor structure of the SOHO-5. Cronbach’s alpha coefficient values for internal consistency were 0.82 for the children’s report and 0.79 for the parental report. The ICCs were 0.90 and 0.89 for the total scores of the children and parental versions in the test-retest reliability analysis. The total SOHO-5 scores for both reports were significantly associated with the global rating questions except for the ’impact on children’s general health’ question in the parental report. Furthermore, the Myanmar version discriminated between the children with and without caries experiences (*p* < 0.001).

**Conclusion:**

This study provided evidence that both children and parental reports of the Myanmar SOHO-5 version have good reliability and validity to assess the OHRQoL of 5-year-old children in a Burmese-speaking population.

## Introduction

Dental caries in children under 6 years old, also called early childhood caries (ECC), represents a major worldwide public health issue and is prevalent in low and middle-income countries [1]. Based on the results of a systematic review, the global prevalence of ECC ranges from 23%–90% [2]. In Southeast Asian countries, the prevalence of ECC among 5–6-year-old children is high with the median caries prevalence at 79% [3]. Untreated caries in deciduous teeth is common, affecting 532 million children worldwide and its prevalence peaks in 5-year-old children [4]. Untreated caries can affect children’s growth, development, and negatively impact their oral health-related quality of life (OHRQoL) [5, 6].

Oral health-related quality of life (OHRQoL) is a multidimensional assessment that reflects the patient’s oral function, and psychological and social aspects [7]. The traditional clinical measures can represent the physical condition of the dental caries status, however, cannot reveal the disease’s impact on the psychosocial factors of the affected children [8]. In addition, assessing the health-related quality of life has become essential to evaluate the interventions and appropriately allocate resources for health care services [9].

In recent years, many oral health-related quality of life measures for various children’s ages have been established [10, 11] and there are also developing new measures specifically for children ≤ 6 years old. Currently, most of the OHRQoL measurements for young children have relied on parent proxy reports [12], however, there is increasing evidence that 4–6 year-old children can reliably report their information regarding their general health and quality of life [13]. Although the parent proxy reports have an important role in children’s OHRQoL measurement, it has been demonstrated that it is better to obtain both perspectives if possible for a comprehensive view [14].

In 2012, the Scale of Oral Health Outcomes for 5-year-old children (SOHO-5) was developed to measure the OHRQoL of young children using both the child’s and parental reports [13]. This measure, which was originally developed in the UK, has been cross-culturally adapted and validated in Bengali [9], Brazilian Portuguese [15], Chinese [10], Indonesian [11], Persian [16], and Spanish languages [17]. These studies found that the adapted SOHO-5 measure is simple, can discriminate between groups with different caries severity, demonstrate satisfactory psychometric properties in other cultures, and is a reliable tool to use for young children.

The Republic of the Union of Myanmar is one of the developing countries where people’s awareness of oral diseases is still low [18]. The first national oral health survey (2017) reported that the mean dmft of 6-year-olds is 5.7 and the untreated caries prevalence is 84.1% [19]. Moreover, no study has measured the OHRQoL in Myanmar young children and there are no validated self-reported OHRQoL scale measurement tools for young children in the Burmese language which is the official language of Myanmar. Therefore, the aim of this study was to perform a cross-cultural adaptation of SOHO-5 to the Burmese language and to investigate its reliability and validity in 5-year-old children.

## Materials and methods

The SOHO-5 consists of a child self-report and a parental report. Both versions contain 7 items. In the child report, the items are ’difficulty in eating, drinking, speaking, playing, sleeping, avoid smiling due to pain, and avoid smiling due to appearance’ with the responses of a 3-point scale (no = 0, a little = 1 and a lot = 2). The items in the parental report comprise ’difficulty in eating, playing, speaking, sleeping, avoid smiling due to pain, avoid smiling due to appearance and affected self-confidence’ with the responses on a 5-point scale (n = 0, a little = 1, moderate = 2, a lot = 3, and a great deal = 4). The SOHO-5 scores for both versions are calculated as the sum of the response codes and a higher score indicates a greater degree of oral impact on the children’s quality of life.

### Cross-cultural adaptation

We developed the SOHO-5 Myanmar version under the permission of the original developer. The original SOHO-5 English version was first translated into Burmese by two bilingual Myanmar translators based on the guidelines for the cross-cultural adaptation process [20]. The conceptual and item equivalence of this translation was revised and evaluated by an expert panel to develop the draft. The panel consisted of a Myanmar pediatric dentist, a dental public health researcher, and a researcher who was familiar with the quality-of-life questionnaire. This draft was translated back into English by two other Myanmar dentists who were blinded to the original instrument. The semantic equivalence between the back-translated version and the original version was compared by the panel that developed the second draft after the revision was performed.

The draft Myanmar version was pilot-tested on 20 pairs of 5-year-old children and their parents to examine the comprehensibility of the Myanmar SOHO-5 version. Based on the feedback, the final Myanmar SOHO-5 version was revised by the expert panel that approved the questionnaire for children and their parents.

### Validity and reliability

This cross-sectional study was conducted in Mandalay, Myanmar, and approved by the Human Research Ethics Committee, Chulalongkorn University (HREC-DCU 2021–047) and the Research and Ethics Committee, Myanmar (ERC-F4-2021).

The study population comprised 5-year-old children and their parents living in the subdistricts of Mandalay. The sample size was calculated according to the internal consistency test (Cronbach’s alpha statistics) using a formula recommended by a previous study [21]. The expected value of Cronbach’s alpha as 0.80 with a 5% precision value and type I error as 0.05 were set for the questionnaire with seven items. The calculation indicated that a minimum of 166 children-parent pairs were needed in this study. Five-year-old children from nine kindergarten schools in seven districts and their parents were invited to participate in this study. An invitation letter with the purpose and procedure of the study was sent to the parents. Written parental consent was obtained prior to implementing the study. Children who were 5 years old, Burmese speaking, and whose parents could understand Burmese were recruited for this study. Children with developmental delays, and disabilities, who were uncooperative, or refused the oral examination were excluded. The SOHO-5 questionnaires for the child and the parental report were completed by conducting face-to-face interviews separately in the classroom. The interviews were performed by three trained research assistants before the oral examination.

The structural validity of the SOHO-5 was examined by using the confirmatory factor analysis (CFA). In this study, construct validity was assessed by examining convergent and discriminant validity. Construct validity revealed the extent to which questions reflect the objectives of the study or not [22]. As a convergent validity, the association between SOHO-5 scores and additional global rating questions for child oral health status was examined in both children’s and parents’ questionnaires.

In the children’s report, the children were asked two additional global rating questions: satisfaction with their oral health (’How happy are you with your teeth? Not happy = 2, A little happy = 1, and very happy = 0’), and the presence of dental cavities (’Do you have any holes in your teeth?; No = 0, Yes = 1’). In the parent’s report, four additional questions were included: Proxy-rated child’s oral health (’How would you rate your child’s dental health?; excellent = 0 to poor = 4’), satisfaction with their child’s oral health (’How happy are you with your child’s dental health?; very happy = 0 to very unhappy = 4’), the child’s overall well-being (’Do you think the overall well-being of your child is affected by the condition of their teeth?; not at all = 0 to a great deal = 4’), and the child’s perceived dental treatment needs (’Do you think your child needs any dental treatment because of the state (holes in teeth or pain) of his/her teeth?; No = 0, Yes = 1’). The difference between the total SOHO-5 score of children with caries and children without caries was assessed for discriminant validity. A second interview was performed on 50 children and their parents 1–2 weeks after the first interview by the same research assistant for the test-retest reliability.

One dentist performed oral examinations of the children in the kindergarten classroom (Kappa: 0.90 for intra-examiner reliability). A disposable dental mirror, a penlight, and a community periodontal index (CPI) probe with a 0.5 mm ball-end were used in the examination. The number of decayed, missing, and filled teeth (dmft index) were recorded for their caries experience following the WHO oral health survey recommendation [23].

### Data analysis

The factor structure was investigated through the confirmatory factor analysis (CFA) using the AMOS statistical package. Three fit indices, the comparative fit index (CFI), the root mean square error of approximation index (RMSEA), and the Tucker–Lewis index (TLI), were used to assess the goodness-of-fit of the model. The model was considered acceptable or a good fit if the values of indices were RMSEA <0.05, CFI ≥0.95, and TLI ≥0.95. The reliability of the Myanmar SOHO-5 measure was assessed by analyzing its internal consistency and test-retest reliability. The internal consistency was assessed by Cronbach’s alpha, as well as Cronbach’s alpha if item deleted and corrected item-total correlation coefficients. The intra-correlation coefficient (ICC) was assessed to measure the agreement level between the responses to the first and second questionnaires. Construct validity was tested by analyzing the association between the Myanmar SOHO-5 scores and the responses to the global rating questionnaires using Spearman’s correlation coefficients. The discriminant validity was examined by comparing the differences between the SOHO-5 scores of children with caries (dmft = 0) and children without caries experiences (dmft > 0) using the Mann-Whitney U-test. The IBM SPSS Statistics analytical software version 26 was used to perform the data analysis.

## Results

The participants in this study comprised 173 children and their parents. The participants consisted of 53.8% boys (n = 93) and 46.2% girls (n = 80). The prevalence of dental caries (dmft > 0) in the study population was 85.5% and their mean dmft was 5.2 (SD = 4.6). The children’s Myanmar SOHO-5 scores ranged from 0–11, with a mean of 1.9 (SD 4.6). The parents’ scores ranged from 0–16, with a mean of 2.8 (SD 3.1).

More than 62% of the children and 67% of the parents reported an oral impact on the quality of life of the children (SOHO-5 scores > 0). ’Difficulty in eating’ was the most reported item, followed by ’difficulty in sleeping’ in children report (S1 Table) and parent report (S2 Table).

**Fig 1:**
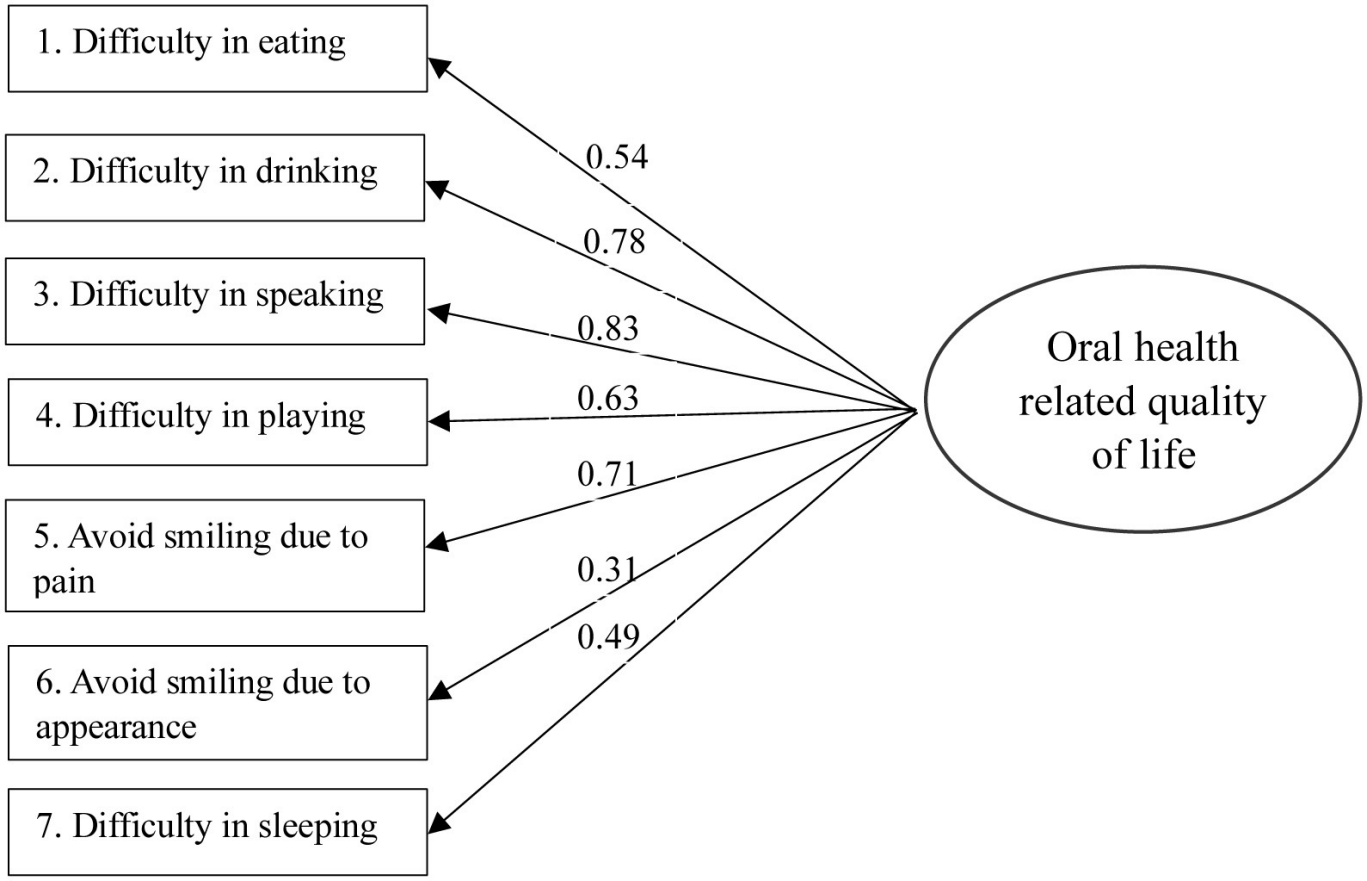
Model for the confirmatory factor analysis (Children version).

The nonsignificant Chi-square value of CFA supported the one-factor model for SOHO-5 in the children version with a Chi-square value of 12.02 with 9 degrees of freedom (*p* = 0.21) as well as in the parent version with a Chi-square value of 13.46 with 12 degrees of freedom (*p* = 0.34). The three fit indices demonstrated the good model fit the data in the children version (CFI = 0.99 > 0.95; RMSEA = 0.04 < 0.05; TLI = 0.98 > 0.95;) and in the parent version (CFI = 0.99 > 0.95; RMSEA = 0.03 < 0.05; TLI = 0.99 > 0.95;). All seven items showed factor loadings higher than 0.30 in the children’s version (Fig 1) and the parent version (S1 Fig).

The overall Cronbach’s alpha coefficients were 0.82 for the children’s and 0.79 for the parental version, respectively, indicating good internal consistency (Table 1). For the test-retest reliability, the ICCs were 0.90 and 0.89 for the total scores of the children and parental versions, respectively, which demonstrated good reproducibility (Table 1).

**Table 1:** Reliability analysis and item characteristics in children.

Item	Internal consistency reliability		Test-retest reliability		
	CITC	Cronbach’s alpha if the item deleted	ICC	95%CI.	*p-value*
**Child version (0–14)**					
Difficulty in eating	0.67	0.78	0.96	0.94–0.98	<0.001
Difficulty in drinking	0.59	0.79	0.69	0.51–0.81	<0.001
Difficulty in speaking	0.65	0.78	0.45	0.20–0.65	<0.001
Difficulty in playing	0.55	0.80	0.56	0.34–0.73	<0.001
Difficulty in sleeping	0.59	0.79	0.84	0.73–0.91	<0.001
Avoid smiling due to pain	0.70	0.76	0.70	0.53–0.82	<0.001
Avoid smiling due to appearance	0.33	0.82	0.64	0.45–0.78	<0.001
**Total scores**	**Cronbach’s alpha 0.82**		**0.90**	**0.83–0.94**	**<0.001**
**Parental version (0–28)**					
Difficulty in eating	0.73	0.73	0.92	0.87–0.96	<0.001
Difficulty in speaking	0.61	0.75	0.67	0.48–0.80	<0.001
Difficulty in playing	0.55	0.77	0.48	0.24–0.67	<0.001
Difficulty in sleeping	0.67	0.72	0.83	0.72–0.90	<0.001
Avoid smiling due to pain	0.64	0.73	0.74	0.58–0.85	<0.001
Avoid smiling due to appearance	0.28	0.79	0.57	0.35–0.73	<0.001
Influence self-confidence	0.41	0.78	0.60	0.39–0.75	<0.001
**Total scores**	**Cronbach’s alpha 0.79**		**0.89**	**0.82–0.94**	**<0.001**

CITC–Corrected item-total correlation, ICC–Intraclass correlation coefficient, CI–Confidence interval.

The construct validity of the children’s version demonstrated that the total SOHO-5 scores were significantly correlated in the expected direction with the two global rating questions, i.e., satisfaction with their oral health (*r* = - 0.71, *p* < 0.001) and self-reported caries (*r* = 0.62, *p* < 0.001) (Table 2).

**Table 2:** Construct validity of the child’s SOHO-5.

Item	Satisfaction with oral health		Self-reported caries	
	*r*	*p-value*	*r*	*p-value*
Difficulty in eating	-0.68	<0.001	0.62	<0.001
Difficulty in drinking	-0.28	<0.001	0.20	0.007
Difficulty in speaking	-0.33	<0.001	0.22	0.003
Difficulty in playing	-0.32	<0.001	0.18	0.017
Difficulty in sleeping	-0.53	<0.001	0.42	<0.001
Avoid smiling due to pain	-0.41	<0.001	0.31	<0.001
Avoid smiling due to appearance	-0.37	<0.001	0.17	0.028
**Total score**	**-0.71**	**<0.001**	**0.62**	**<0.001**

*r—*Spearman’s correlation coefficient.

In the parental version, the total SOHO-5 scores were significantly correlated with the three respective questions, i.e., parent-rated oral health (*r* = 0.77, *p* < 0.001), satisfaction with the child’s oral health (*r* = 0.76, *p* < 0.001) and the child’s perceived treatment need (*r* = 0.75, *p* < 0.001) (Table 3).

**Table 3:** Construct validity of the parent’s SOHO-5.

Item	Parent-rated oral health		Satisfaction		Impact on general health		Treatment need	
	*r*	*p-value*	*r*	*p-value*	*r*	*p-value*	*r*	*p-value*
Difficulty in eating	0.77	<0.001	0.76	<0.001	0.13	0.096	0.72	<0.001
Difficulty in speaking	0.33	<0.001	0.38	<0.001	0.22	0.003	0.26	<0.001
Difficulty in playing	0.31	<0.001	0.39	<0.001	0.16	0.031	0.20	0.008
Difficulty in sleeping	0.59	<0.001	0.62	<0.001	0.17	0.022	0.54	<0.001
Avoid smiling due to pain	0.43	<0.001	0.45	<0.001	0.02	0.771	0.39	<0.001
Avoid smiling due to appearance	0.30	<0.001	0.30	<0.001	-0.004	0.957	0.257	0.001
Influence on self-confidence	0.25	0.001	0.25	0.001	0.05	0.506	0.33	<0.001
**Total score**	**0.77**	**<0.001**	**0.76**	**<0.001**	**0.09**	**0.225**	**0.75**	**<0.001**

*r—*Spearman’s correlation coefficient.

For the discriminant validity, the children with a history of dental caries had a higher mean rank of SOHO-5 total scores compared with children without caries experiences in the children’s reports (96.12 vs. 33.00, *p* < 0.001 and parental report (96.61 vs. 30.08, *p* < 0.001) (Tables 4 and 5).

**Table 4:** Discriminant validity of the child’s SOHO-5.

Item	Mean ranks		P value
	*Caries free*	*Caries*	
Difficulty in eating	35.00	95.78	<0.001
Difficulty in drinking	79.00	88.35	0.085
Difficulty in speaking	77.50	88.60	0.059
Difficulty in playing	80.50	88.10	0.125
Difficulty in sleeping	60.50	91.48	<0.001
Avoid smiling due to pain	65.50	90.63	0.002
Avoid smiling due to appearance	76.00	88.86	0.040
**Total score**	**33.00**	**96.12**	**<0.001**

Mann-Whitney U test.

**Table 5:** Discriminant validity of the parent’s SOHO-5.

Item	Mean ranks		P value
	*Caries free*	*Caries*	
Difficulty in eating	32.00	96.29	<0.001
Difficulty in speaking	76.50	88.77	0.045
Difficulty in playing	80.50	88.10	0.125
Difficulty in sleeping	54.50	92.49	<0.001
Avoid smiling due to pain	64.50	90.80	0.002
Avoid smiling due to appearance	77.00	88.69	0.052
Influence on self-confidence	70.88	89.72	0.017
**Total score**	**30.08**	**96.61**	**<0.001**

Mann-Whitney U test.

## Discussion

The present study performed a cross-cultural adaptation of SOHO-5 to the Burmese language and evaluated its reliability and validity in 5-year-old children. The results demonstrated that the Myanmar SOHO-5 version was successfully developed, and its psychometric properties were acceptable for Myanmar 5-year-old children. A standard cross-cultural adaptation procedure was followed to ensure that all the items in the child’s and parent’s reports were retained in the Myanmar SOHO-5 version.

In this study, the 5-year-old children understood the content of the SOHO-5 and responded appropriately to the questions. The children’s and parents’ versions reported that ~60% of the children in the sample population had an oral impact on their daily life with SOHO-5 > 0. Therefore, it also revealed that the children could report their own perceptions of their OHRQoL, and studies on the OHRQoL of children should not only depend on the parental proxy reports.

Regarding the factor structure, the confirmatory factor analysis indicated the proposed one-factor structure of the SOHO-5 measure. A good fit of the model supported the information about the unidimensional characteristic of the SOHO-5 instrument for the OHRQoL similar to the previous finding [17]. Although some factor loadings were lower than those of the previous finding, the results indicated that the test items have acceptable psychometric properties. Moreover, the item of “Avoiding smiling due to appearance” was lower factor loading than other items, it might be this item was less important issue in their life. When evaluated for reliability, all inter-item correlations were positive, and all corrected item-total correlations were above the minimum recommended level of 0.20 for including an item in a scale, which indicated the items in the SOHO-5 scale were correlated conceptually [24]. Furthermore, the child’s and parent’s versions had a standardized Cronbach’s alpha above the recommended level of 0.70 indicating good internal consistency, which was similar to the results of the original study and studies in other populations [25]. The value of the total Cronbach’s alpha did not improve when any of the items were deleted, however, after deleting the item ’avoid smiling due to appearance’; the Cronbach’s alpha values for both versions were the same as the total alpha values. However, this result is not sufficient justification to remove this item from the Myanmar SOHO-5 while the other results demonstrated good performance. The ICC values of the child’s and parent’s reports in this study presented a high degree of agreement between the scores at different times, which reflected the excellent test-retest reliability.

The construct validity results indicated that the associations between the total SOHO-5 scores and the different subjective global rating questions of the child’s and parent’s reports were significant in the expected direction, however, the children’s general health question in the parent’s version had a significant correlation in some items. This finding regarding the impact on children’s general health was similar to studies in Indonesia and may be due to the parent’s underestimation of the impact of oral health on the overall well-being of the children. A possible reason is the lack of people’s awareness of oral health due to an insufficient number of dental professionals, limited oral health promotion activities, and oral health care services in Myanmar [26]. However, these consistent findings indicated good construct validity for both versions of this measure. Moreover, the total SOHO-5 scores of both reports were significantly higher in children with caries, which demonstrated the discriminant ability of the measures between children with and without caries experiences.

When consider as the criteria for good measurement properties tool of the Consensus-based Standards for the selection of health Measurement INstruments (COSMIN) [22], this Myanmar version followed all the steps according to the guideline for the process of cross-cultural adaptation. For the assessment of psychometric properties, this version assessed 1) structural validity; 2) internal consistency determined by Cronbach’s alpha; 3) reliability demonstrated by the intraclass correlation coefficient (ICC); 4) hypothesis testing for construct validity. However, Myanmar where English is not frequently used in addition to the native language and where bilingualism is not common, therefore it cannot assess the cross-cultural validity. Moreover, due to the limitation of the cross-sectional study design and further longitudinal studies are required to examine the properties of the measurement error and the responsiveness of SOHO-5.

Myanmar SOHO-5 is the first measurement tool in the Myanmar language to assess the OHRQoL of preschool children through their self-report. Tooth decay in young children in Myanmar is a public health issue. In Myanmar, dental manpower and resources are limited and national programs to tackle the burden of ECC are not yet prioritized [27]. Using the Myanmar SOHO-5 in addition to oral health surveys or project evaluation will be beneficial for health policymakers and health workers where oral diseases in children are high and left untreated in Myanmar. The limitation of the study should be also addressed. Many studies reported the possible limitations of the dmft index used in caries assessment such as the under or overestimation of the carious lesion, not related to the teeth at risk [28]. It is recommended to use the ICDAS in caries assessment to identify the severity of carious lesions in future studies and considering the presence of pain is one of the most disabling oral conditions more than dmft which is not a good indicator of the severity of the decay. Although Burmese is the national language and the most commonly used language in Myanmar, there are several ethnic minorities who speak various dialects in Myanmar. Thus, the Myanmar SOHO-5 might not be able to be used in some areas where young children do not speak Burmese well. Further research on assessing the OHRQoL of children using a representative sample with a wide range of socio-economic positions is recommended.

## Conclusion

This study demonstrates that both Myanmar SOHO-5 versions have good reliability (internal consistency, test-retest reliability) and validity (construct, discriminant validity). Therefore, this instrument was successfully developed and is appropriate to use in the assessment of OHRQoL of 5-year-old children in Myanmar.

## Supporting information

S1 Fig Model for the confirmatory factor analysis (Parental version). (DOCX)

S1 Table Distribution of the child’s SOHO-5 responses. (DOCX)

S2 Table Distribution of the parent’s SOHO-5 responses. (DOCX)

S1 Questionnaire SOHO-5 questionnaire (Myanmar version). (DOCX)
