# Supplementary material for: Cross-cultural adaptation and psychometric properties of the Myanmar version of the scale of oral health outcomes for 5-year-old children
Source: PLoS One. 2023 Mar 22;18(3):e0282880. doi: 10.1371/journal.pone.0282880 (PMC10032510; doi:10.1371/journal.pone.0282880)
Supplement: S1 Fig — (DOCX) [file pone.0282880.s001.docx]

**Figure 1. Model for the confirmatory factor analysis (Parent version)**

1. Difficulty in eating

7. Difficulty in sleeping

5. Avoid smiling due to pain

6. Avoid smiling due to appearance

4. Difficulty in playing

3. Difficulty in speaking

2. Self confidence

0.64

0.45

0.76

0.32

0.61

0.67

0.74

**CFI = 0.99 > 0.95; RMSEA = 0.03 < 0.05; TLI = 0.99 > 0.95;), Chi-square =13.46, df = 12, *p*=0.34**
