# Supplementary material for: Cross-cultural adaptation and psychometric properties of the Myanmar version of the scale of oral health outcomes for 5-year-old children
Source: PLoS One. 2023 Mar 22;18(3):e0282880. doi: 10.1371/journal.pone.0282880 (PMC10032510; doi:10.1371/journal.pone.0282880)
Supplement: S1 Table — (DOCX) [file pone.0282880.s002.docx]

**Table S1. Distribution of the Child’s SOHO-5 responses**

| Item | No (%) | A little (%) | A lot (%) |
| --- | --- | --- | --- |
| Difficulty in eating | 69 (39.9) | 66 (38.2) | 38 (22.0) |
| Difficulty in drinking | 157 (90.8) | 15 (8.7) | 1 (0.6) |
| Difficulty in speaking | 154 (89.0) | 15 (8.7) | 4 (2.3) |
| Difficulty in playing | 160 (92.5) | 10 (5.8) | 3 (1.7) |
| Difficulty in sleeping | 120 (69.4) | 43 (24.9) | 10 (5.8) |
| Avoid smiling due to pain | 130 (75.1) | 38 (22.0) | 5 (2.9) |
| Avoid smiling due to appearance | 151 (87.3) | 21 (12.1) | 1 (0.6) |
