# Supplementary material for: Cross-cultural adaptation and psychometric properties of the Myanmar version of the scale of oral health outcomes for 5-year-old children
Source: PLoS One. 2023 Mar 22;18(3):e0282880. doi: 10.1371/journal.pone.0282880 (PMC10032510; doi:10.1371/journal.pone.0282880)
Supplement: S2 Table — (DOCX) [file pone.0282880.s003.docx]

**Table S2. Distribution of the Parent’s SOHO-5 responses**

| Item | Not at all (%) | A little (%) | Moderate (%) | A lot (%) | A great deal (%) |
| --- | --- | --- | --- | --- | --- |
| Difficulty in eating | 63 (36.4) | 43 (24.9) | 38 (22.0) | 25 (14.5) | 4 (2.3) |
| Difficulty in speaking | 152 (87.9) | 15 (8.7) | 6 (3.5) | 0 | 0 |
| Difficulty in playing | 160 (92.5) | 11 (6.4) | 2 (1.2) | 0 | 0 |
| Difficulty in sleeping | 108 (62.4) | 39 (22.5) | 20 (11.6) | 6 (3.5) | 0 |
| Avoid smiling due to pain | 128 (74.0) | 30 (17.3) | 14 (8.1) | 1 (0.6) | 0 |
| Avoid smiling due to appearance | 153 (88.4) | 17 (9.8) | 3 (1.7) | 0 | 0 |
| Influence self confidence | 134 (77.5) | 35 (20.2) | 4 (2.3) | 0 | 0 |
