# Supplementary material for: Cross-cultural adaptation and psychometric properties of the Myanmar version of the scale of oral health outcomes for 5-year-old children
Source: PLoS One. 2023 Mar 22;18(3):e0282880. doi: 10.1371/journal.pone.0282880 (PMC10032510; doi:10.1371/journal.pone.0282880)
Supplement: S1 Questionnaire — (DOCX) [file pone.0282880.s004.docx]

## Scale of Oral Health Outcomes for 5-years old children (SOHO-5) (Child version)

**အဖြေ။ မရှိပါ အနည်းငယ် များစွာ**

**၁။ အစားစားရာတွင် ခက်ခဲခြင်း**   

**၂။ ရေသောက်ရာတွင် ခက်ခဲခြင်း**   

**၃။ စကားပြောရာတွင် ခက်ခဲခြင်း**   

**၄။ ကစားရာတွင် ခက်ခဲခြင်း**   

**၅။ နာကျင်သောကြောင့် ပြုံး/ရယ်ခြင်းကို ရှောင်သည်**   

**၆။ ပုံသဏ္ဍန်အသွင်အပြင်ကြောင့် ပြုံး/ရယ်ခြင်းကို ရှောင်သည်**   

**၇။ အိပ်စက်ရာတွင် ခက်ခဲခြင်း**   

## Scale of Oral Health Outcomes for 5-years old children (SOHO-5) (Parent version)

**အဖြေ။ လုံးဝ အနည်းငယ် အသင့်အတင့် အတော်များများ များစွာ**

**၁။ အစားစားရာတွင် ခက်ခဲခြင်း**     

**၂။ စကားပြောရာတွင် ခက်ခဲခြင်း**     

**၃။ ကစားရာတွင် ခက်ခဲခြင်း**     

**၄။ နာကျင်သောကြောင့် ပြုံး/ရယ်ခြင်းကို ရှောင်သည်**     

**၅။ ပုံသဏ္ဍန်အသွင်အပြင်ကြောင့် ပြုံး/ရယ်ခြင်းကို ရှောင်သည်**     

**၆။ အိပ်စက်ရာတွင် ခက်ခဲခြင်း**     

**၇။ မိမိကိုယ်ကို ယုံကြည်မှုအပေါ် သက်ရောက်မှုရှိခြင်း**     
